# Recycled Precast Concrete Kerbs and Paving Blocks, a Technically Viable Option for Footways

**DOI:** 10.3390/ma14227007

**Published:** 2021-11-19

**Authors:** Andrés Juan-Valdés, Desirée Rodríguez-Robles, Julia García-González, María Isabel Sánchez de Rojas, Manuel Ignacio Guerra-Romero, Rebeca Martínez-García, Julia M. Morán-del Pozo

**Affiliations:** 1 Department of Agricultural Engineering and Sciences, University of León, Av. Portugal 41, 24071 León, Spain; julia.garcia@unileon.es (J.G.-G.); ignacio.guerra@unileon.es (M.I.G.-R.); julia.moran@unileon.es (J.M.M.-d.P.); 2 Department of Agronomy and Forestry Engineering, University of Extremadura, Av. Adolfo Suárez, s/n, 06007 Badajoz, Spain; desireerodriguez@unex.es; 3 Eduardo Torroja Institute for Construction Science, Serrano Galvache 4, 28033 Madrid, Spain; srojas@ietcc.csic.es; 4 Department of Mining Technology, Topography and Structures, University of León, Campus de Vegazana s/n, 24071 León, Spain; rmartg@unileon.es

**Keywords:** recycled cement, mixed recycled aggregates, recycled concrete, precast concrete, footways

## Abstract

The linear economy paradigm in place to date has to be seriously challenged to give way to a new school of thought known as the circular economy. In this research work, precast kerbs and paving blocks made with recycled concrete (RACC-mixture) bearing 50 wt% mixed recycled aggregate (masonry content of 33%) and an eco-efficient cementitious material as 25 wt% conventional binder replacement were evaluated to assess their intrinsic potential to replace traditional raw materials, in keeping with circular economy criteria. Therefore, precast products were subjected to mechanical strength, durability and microstructure tests and were compared to conventional concrete units (CC-mixture and commercially available precast elements). Although a class demotion was observed for water absorption and some decreases in flexural strength (26%), splitting tensile strength (12.8%) and electrical resistivity (45%) and a lower class water absorption were registered, and the recycled mixture also exhibited a greater performance in terms of compressive strength (6%), a better abrasion resistance classification and a comparable porosity and microstructure, which ensures a good concrete durability. In any case, the results showed that precast pieces were European standard-compliant, thus supporting the viability of the mixed recycled aggregates and eco-efficient cementitious replacement in footways.

## 1. Introduction

Millions of kerbs and pavement blocks are made every year to repair or to build new footways. They are bulk-concrete precast, made usually with natural aggregates. There is a good chance to use recycled concrete in this kind of industrial products. However, so far, there are not many studies that deal with this matter. All of them investigate the effect of using recycled aggregates in its properties [1,2,3,4,5,6,7,8].

At the European Council’s 1998 meeting at Cardiff [9], the EU defined three types of sustainability in line with the provisions of Article 6 of the Treaty on European Union, i.e., to further ‘the integration of environmental protection into Community policies in order to achieve sustainable development’. Whilst global consensus in the matter has not yet been forthcoming, support for a green and sustainable environment is growing.

Against that backdrop, for a number of decades the international scientific community has produced hosts of studies attesting to the potential of secondary aggregates and pozzolanic additions sourced from construction and demolition waste, CDW (Construcition and demolition waste) as replacements for natural aggregate and primary binders, primarily clinker.

Of the various types of recycled coarse aggregates in place, the one where output is highest in many EU countries is so-called mixed recycled aggregate, MRA, in whose composition masonry waste—brick, roof and indoor tiles and similar—accounts for over one-third of the total [10]. Masonry waste also generates fines, for its intrinsic properties such as fired clay make it usable in addition with pozzolanic potential in eco-efficient cements, conventionally manufactured with industrial by-products such as fly ash and blast furnace slag.

Whereas the use of mixed recycled coarse aggregate and CDW masonry—primarily brick—powder has been amply studied, their joint application in recycled concrete manufacturing has been seldom explored by the scientific community [11,12,13,14,15]. Even less research has been conducted into precast products made with recycled concrete, though they have been found to be technically and economically comparable to commercial precast units and hence are viable secondary materials.

In the non-structural bulk concrete formulated and tested here—RACC concrete mixture—50 wt% of the natural aggregate was replaced with mixed recycled aggregate as supplied by a waste treatment plant (i.e., *ex professo* preparation of the secondary aggregates took place); this mixed recycled aggregate could be regarded as a selling point in the future acceptance of its use and could demonstrate a competitive advantage towards the conventional products, as it would surely affect the costs: in other words, as it was not explicitly conditioned or prepared for this study, but the findings revealed its intrinsic potential.

The cement used was also recycled, bearing 75% CEM I and 25% pre-conditioned masonry powder sourced from CDW. The fact that the ceramic by-products have already gone through an activation thermal treatment makes them ideal candidates for reuse as pozzolanic additions, without having to incur additional energy consumption, which increases the sustainability of this reutilisation alternative.

The units studied, kerbs and paving blocks, are extensively used in construction, primarily for footways. Commercially available products made with a natural aggregate and blast furnace slag-bearing cement (a CC concrete mixture) were compared to recycled concrete units made with mixed recycled aggregate and eco-efficient cement containing masonry powder sourced from CDW, since those are the products that eventually the recycled precast pieces would have to substitute.

The purpose of the study was to ascertain the potential mechanical strength and durability of recycled kerbs and paving blocks bearing secondary raw materials drawn from construction and demolition waste as an ideal replacement for traditional raw materials, with a view to envisaging a more responsible and environmentally sustainable future for the construction industry.

## 2. Materials and Methods

### 2.1. Natural Aggregates

The natural aggregates applied here (supplied by Prefabricados Páramo, S.L. and consisting in 0/4 mm ground and 0/5 mm riverbed sand and 6/12 mm and 4/10 mm gravel) are ordinarily used to manufacture non-structural precast concrete paving blocks, kerbs, pan forms and similar. The aim was to prepare a control concrete with those materials as comparable as possible to the recycled concrete formulated in this study.

The concrete was made with fine and coarse aggregate, both siliceous, conforming to the specifications on aggregates apt for use in concretes set out in the Spanish Structural Code [16] and European standard EN 12620:2003+A1 [17].

**Figure 1:**
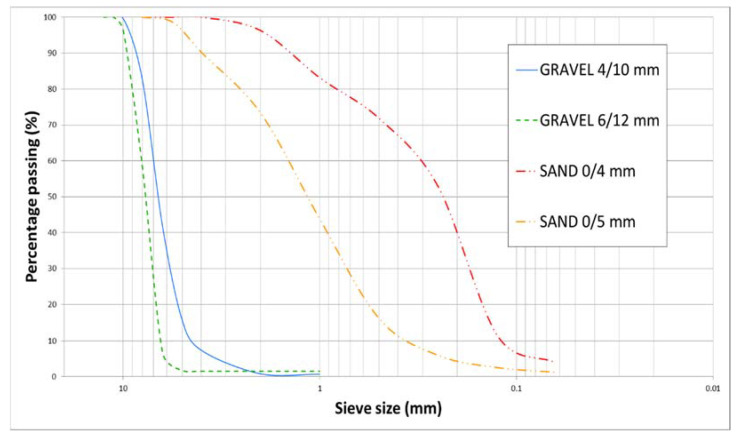
Particle size distribution of the natural (fine and coarse) aggregates used in the study.

The particle size distribution for those materials, determined further to European standard EN 933-1 [18], is plotted in Figure 1.

The chemical composition of the natural aggregates found with X-ray fluorescence (XRF) is given in Table 1, which attests to their high siliceous content.

### 2.2. Recycled Aggregates

The mixed recycled aggregates used in this study were obtained by mechanically separating, crushing and sieving construction and demolition waste at the TEC-REC: Tecnología y Reciclado S.L. CDW management plant located in the province of Madrid, Spain.Their EN 12620:2003+A1 [17]-determined physical and mechanical properties are summarised in Table 2.

The non-floating components in the recycled aggregate listed in Table 3 were determined as specified in standard EN 933-11 [24].

### 2.3. Conventional Cement

The chemical composition of the European and Spanish standard (EN 197-1 [25]; UNE 80303-1 [26])-compliant CEM III/A 42.5 N/SR commercial cement (blended with blast furnace slag) used in this study is compared to European legislation specifications in Table 4.

Cement choice depends heavily on the characteristics of any given construction project or application, including requirements on strength, durability, high early age strength, sulfate resistance, limits to tricalcium aluminate content, heat of hydration, concrete fineness to prevent shrinkage and similar. According to some authors and studies, CEM III is recommended for use with recycled aggregates, inasmuch as its fly ash or blast furnace slag additions prevent alkali-aggregate reactions in concretes bearing recycled aggregates [27].

The strength of the conventional or ordinary cements most commonly used to manufacture recycled concrete is typically 42.5 MPa. Sulfate-resistant cement is also beneficial, for it minimises the risk identified by some authors of the reaction between the tricalcium aluminate hydrate in the hardened paste with external sulfates, which could prompt sulfoaluminate formation and the concomitant rise in volume.

Blast furnace slag-bearing cement was chosen here based on earlier findings [8,28,29] and recommendations set out in Mas et al. [30]

### 2.4. Recycled Cement

The recycled cement used in this study was designed by researchers at the Eduardo Torroja Institute for Construction Science. Given the pozzolanicity of the fired clay materials commonly and extensively used to manufacture masonry products such as brick and roof tiles and their removal to construction and demolition waste heaps at the end of their service lives, those authors tested their use as alternative additions in cementitious binders. The pozzolanic powder used as an addition had been characterised in earlier studies [31,32,33,34,35,36] to determine its sulfate resistance and calorimetry during cement hydration.

The macroscopic composition of the masonry waste deployed, sourced from a management plant in the Spanish province of León, was 100% fired clay. Prior to use it was pre-conditioned by drying at 105 C to a constant weight, crushed in a jaw crusher, ground in a ring grinder and sieved to <63 μm. That powder was subsequently blended with ordinary portland CEM I 42.5 N cement in a shaker-mixer. The X-ray fluorescence (XRF)-determined chemical composition of the masonry powder is given in Table 5, which shows, as expected, that the major component was silicon oxide (SiO_2_), followed by aluminium (Al_2_O_3_) and iron (Fe_2_O_3_) oxides.

The chemical composition of the resulting recycled eco-efficient cement, a blend of 75% CEM I and 25% clay brick powder, is listed in Table 6.

Although the use of certain cement additions is envisaged in EN 197-1 [25], that European standard also lays down a series of mechanical, chemical and physical requirements that must be met by such materials, including minimum compressive and flexural strength, maximum SO_3_ and Cl^−^ content, minimum initial and final setting times and soundness. Further to the values for those parameters (Table 7) the eco-efficient cement applied here was EN 197-1-compliant in those respects. The findings also corroborated the lower environmental impact of using clay brick powder derived from CDW.

The advantage of this type of cement is the lower energy cost of cement manufacture, for the (previously fired) clay, added during cement manufacture, does not have to be burnt in the cement kiln and therefore consumes no energy for desiccation, dehydration or calcination.

### 2.5. Water

Further to Spain’s Structural Code [16], the clean and impurity-free water must be used to mix cement to prevent variations in cement hydration, setting or hardening delays or declines in strength or durability. Its purpose, in addition to hydrating the cement and other active components, is to lubricate the mix to ensure fresh-state workability and create voids in the paste to house cement hydration products.

The concretes in this study were mixed with potable, Structural Code [16]-compliant tap water.

### 2.6. Conventional and Recycled Concrete Batching

Concrete batching calls for a detailed study of the characteristics of the components, given the highly significant effect of their inter-relationships on mix behaviour. Design requirements for the specific project where the concrete is to be used must also be scrupulously honoured. Here the concrete was intended for non-structural precast elements conforming to the mechanical and durability requirements set out in the European (EN) standards governing paving block and kerb production.

Bearing in mind the basic batching for commercial precast concrete and earlier findings [18,19,20], the batching used was as listed in Table 8. The aim was to ensure a characteristic strength of 25 MPa in both the conventional and the recycled concrete prepared using the same w/c ratio to ensure comparability. In keeping with routine practice [39,40], coarse aggregate replacement was mass- (rather than volume-) based.

### 2.7. Precast Units

Precast monoblock kerbs and pavers were manufactured in the laboratory as specified in Spanish standard UNE 127340 [41] and European standard EN 1338 [42,43]. The experimental units were prepared as per EN 12390-1 [44] and EN 12390-2 [45]. After the concrete was poured into moulds and the exposed surfaces smoothed to ensure a suitable finish, the units were stored in plastic film for 24 h, when they were removed from the moulds for curing.

In keeping with the dimensions routinely found in Spanish class A2 urban footways (standard UNE 127340 [41]), the kerbs were sized to a length of 1000 mm and a cross-section of 200 mm × 100 mm.

The paving blocks were likewise dimensioned to routine practice in Spanish urban settings, i.e., to a cross-section of 100 mm × 80 mm cross-section and a length of 200 mm.

### 2.8. Methods

#### 2.8.1. Geometric and Visual Requirements

The geometric/dimensional tolerance, surface finishes and visual (appearance, texture, colour) characteristics of the units were verified against the specifications laid down in standards EN 1340 [46,47] and UNE 127340 [41] for kerbs and in EN 1338 [42,43] for paving blocks.

#### 2.8.2. Compressive Strength

Both recycled and conventional concrete mixtures were assessed on the laboratory hydraulic press. Standard 150 mm × 300 mm cylindrical specimens conforming to [44] were tested in uniaxial compression at different ages (7 d, 21 d and 28 d) for compressive strength as described in EN 12390-3/AC [48].

#### 2.8.3. Kerb Flexural Strength

Eight recycled precast kerb units (200 mm × 100 mm × 1000 mm) were tested for 28 d flexural strength in keeping with standards EN 1340 [46,47] and UNE 127340 [41]. Results from commercially available precast kerbs were used to compare conventional versus recycled precast elements.

#### 2.8.4. Paving Block Splitting Tensile Strength

Eight 28 d recycled paving blocks (100 mm × 80 mm × 200 mm) were tested for splitting tensile strength as laid down in standard EN 1338 [42,43]. RACC versus CC comparison was made based on the strength of the results of commercially available precast paving blocks.

#### 2.8.5. Abrasion Resistance and Water Absorption

Abrasion resistance was evaluated as per standards EN 1340 [46,47] and UNE 127340 [41] on three specimens of both paving blocks (100 mm × 80 mm × 200 mm) and kerbs (200 mm × 100 mm × 1000 mm) made with the RACC concrete mixture, whereas the results from CC mixtures correspond to commercially available elements.

#### 2.8.6. Water Absorption

Three paving blocks (100 mm × 80 mm × 200 mm) and kerbs (200 mm × 100 mm × 1000 mm) RACC concrete were tested for water absorption according to standard EN 1338 [42,43]. Moreover, results from commercially available precast elements were used for comparison purposes.

#### 2.8.7. Electrical Resistivity

CC and RACC concrete electrical resistivity was found at 20 °C on 100 mm × 200 mm saturated surface-dry cylindrical specimens in keeping with the protocols set out in standard UNE 83988-1 [49]. To ensure a proper electrical contact, two wetted sponges (5 mm thickness) of known electrical resistance were inserted in between.

#### 2.8.8. Microstructure

Concrete microstructure was analysed on a Hitachi S-4800 (Hitachi Group, Tokyo, Japan) scanning electron microscope (SEM) fitted with a tungsten X-ray emitter and Si/Li detector and coupled to a Brucker XFlash 5030 EDS analyser. All samples were mounted on a metal sample holder for readier placement in the microscope and carbon-coated to ensure conductivity and avoid signal masking.

#### 2.8.9. Porosity

This technique, which yields information on material pore structure, is based on the capillarity generated in liquids that do not soak adjacent solids. A liquid such as mercury with a 141° contact angle against a solid surface can only be inserted into the material pores if the pressure applied is greater than that exerted by the capillarity. Porosity tests on 28-day cured RACC concrete samples (20 mm diameter and 20 mm height extracted from a standard cylindrical specimen) were conducted on a Micromeritics (Norcross, GA, USA) 9500 mercury porosimeter featuring a pressure range for mercury intrusion of 0.00345 MPa to 227.53 MPa. To ensure moisture removal, the samples were previously dried to a constant weight at 40 °C and degasses with a vacuum pump (30 min).

## 3. Results

### 3.1. Geometric and Visual Requirements

Both concretes, CC and RACC, met the dimensional requirements for paving blocks and kerbs laid down in the respective European standards [42,43,46,47]. As this study was based on units commercialised by a precasting company, the manufacturers’ dimensions were used as a reference, i.e., the dimensions against which the laboratory-prepared elements were verified to ensure they lay within the tolerances specified. None of the units made with RACC were observed to lie outside the tolerance limits, with the widest deviation (−3 mm) found in the height of one of the paving blocks. Neither the use of mixed recycled aggregate in the concrete or of masonry waste in the cement in the proportions tested in this study compromised compliance with the dimensional requirements for paving blocks or kerbs. The commercial precast elements made with CC, in turn, likewise exhibited dimensions within the allowable range, a finding consistent with the strict quality control enforced during their manufacture.

**Figure 2:**
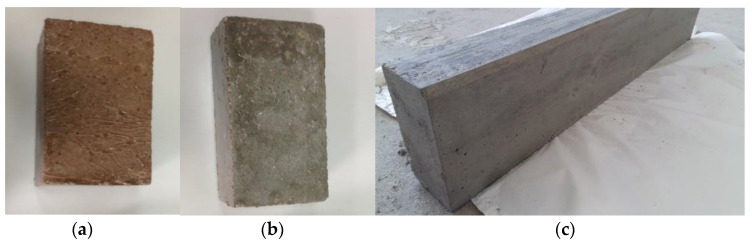
Precast units: (**a**) RACC paving block; (**b**) CC paving block; (**c**) CC kerb.

The visual appearance, texture and colour of the two materials were also compared. No surface cracking or flaking was observed in any of the units, irrespective of the material used in their preparation. No significant textural differences were perceived between the CC and RACC specimens. Colour differences were readily detected, however (Figure 2), for the elements made with RACC exhibited a reddish tone attributable to the colour of the brick used to manufacture the recycled cement.

Despite that obvious visual difference between the two concretes, the use of RACC should not be ruled out on those grounds only. Rather, it should be valued for its potential as a sustainable alternative to dying precast elements, in keeping with routine practice in the commercial manufacture of urban footway paving blocks.

### 3.2. Mechanical Strength Requirements

The strength values for CC and RACC are given in Table 9.

#### 3.2.1. Compressive Strength

**Figure 3:**
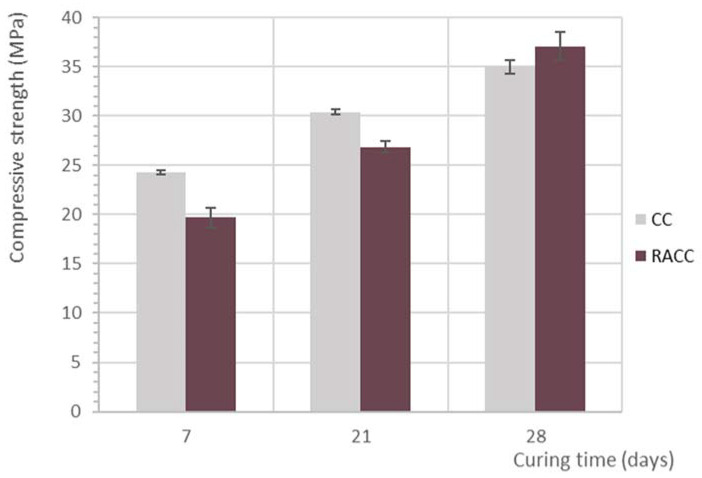
CC and RACC characteristic compressive strength by curing age.

Compressive strength in CC and RACC is plotted against time in Figure 3.

At the earlier ages, CC exhibited higher compressive strength than RACC (Table 9), with values 19% lower in the 7 d and 12% lower in 21 d RACC. Similar findings have also been reported in the literature on concretes made with an recycled aggregate [50,51,52] and other papers on studies of concretes made with coarse aggregate and recycled cement [14]. The less intense decline in strength over time denotes differences in the mechanical development between conventional concrete and recycled materials containing masonry waste both as coarse aggregate and a cement addition. Such behaviour may be attributed to a number of factors:

- The filler effect and pozzolanicity of recycled brick powder-based cement additions. The findings reported by Olofinnade et al. [53] endorse the benefits of brick powder as a partial cement replacement for concrete compressive strength. Other studies [13,54,55,56,57], however, in light of adverse effects observed at high replacement rates, propose 15% to 30% limits to addition content. Ma et al. [58] contend that compressive strength declines with rising replacement ratios when the specific surface of the brick powder used is similar to or lower than in cement.
- The presence of unhydrated cement particles in the recycled aggregate, which may induce secondary hydration.
- The effect of internal curing prompted by non-compensation for the water absorbed by recycled materials such as aggregates [59] or cement additions [60], from which the water initially absorbed is gradually returned to the concrete mix.

The CC exhibited 28 d compressive strength of 35 MPa and the RACC 37.1 MPa (Table 9), substantially higher than the dosage target value and comfortably above the 20 MPa specified for structural use in standard EN 1992-1-1 [61,62]. In contrast to the findings for the earlier ages, here the RACC had 6% higher compressive strength than the reference concrete, attributable to:

- the pozzolanicity of the recycled materials used, which has even been shown to effectively prevent strength loss induced by the use of recycled aggregates [63]. Moreover, the specific surface of the brick powder applied here (5735 cm^2^/g) was higher than the 3593 cm^2^/g, characterising the CEM III/A found in the reference concrete, whilst the replacement ratio defined lay within the range recommended in the literature.
- The absence of any technique to offset water absorption, lowering the effective w/c ratio and favouring higher mechanical performance in the RACC.
- The strength of the interfacial transition zone (ITZ) between the recycled aggregate and the cement paste, as per the microstructural analysis discussed below.

The present findings contrast with those of other research published in the existing literature for the same replacement ratios (50% RA and 25% masonry addition), where compressive strength was reported to decline. In an initial study, Cantero et al. [14] recorded a 19.5% decline in 28 d recycled concrete mechanical strength relative to the reference, whilst Letelier et al. [13] observed losses of 11% to 13% where recycled concrete aggregate and brick powder were used. Those authors also noted that the difference narrowed with the curing time to values of −7.4% after 1 year. Lower performance in the materials bearing dually valorised masonry CDW was consequently attributed to the longer hydration time required by recycled cement [33]. In a second study (Cantero et al., 2020a), the aforementioned authors observed even steeper 28 d declines, at 50% for cubic and 57.2% for cylindrical specimens. The nature of their experiment enabled them to identify the poor quality of the recycled coarse aggregate as the factor primarily involved in lowering compressive strength.

#### 3.2.2. Kerb Flexural Strength

Aggregate shape is known to have a significant impact on concrete flexural strength. The jaggedness and surface roughness typical of recycled aggregate may be expected to have a beneficial effect on recycled concretes. Whereas some authors have reported comparable strength for recycled and conventional aggregate [64,65,66] or only minor differences (up to 10%) in favour of the latter [5,7,8,52,67,68,69], many others have observed up to 45% lower values in the recycled than in the reference concrete [3,50,51,59,70,71,72].

The authors of a number of studies found flexural strength to be similar in concrete manufactured with conventional and blended (brick powder-additioned) cement, especially where low replacement ratios were applied: 10% [12], 15% [11,13] and even up to 30% [55].

In a subsequent study, Ge et al. [55] indicated that the effect of masonry additions, which rose with the replacement ratio in the 10% to 30% range studied and was significant in 7 d materials, with declines of up to 67.63% for the 30% additioned concrete relative to the reference. The same authors reported that the addition had no significant effect in the 28 d materials, irrespective of the replacement ratio. Along those lines, although in connection with mortars bearing masonry additions, Naceri and Hamina [73] observed differences in 7 d and 28 d flexural strength, whereas the 90 d findings for the reference and 10% additioned materials were comparable. More recently, Ma et al. [58] also detected differences in 28 d mortar compressive strength, which widened with higher masonry addition replacement ratios.

Further to the data in Table 9, the kerbs tested here for flexural strength yielded values of 5 MPa (CC) and 3.7 MPa (RACC). The use of 50% mixed recycled aggregate to replace conventional gravel, together with the addition of 25% masonry powder to the recycled cement, were therefore observed to have an adverse effect, with a decline on the order of 26% in recycled concrete performance. In other words, the RACC performed less well in terms of flexural than in terms of compressive strength. The findings recorded here carried no indication of the beneficial effects of masonry addition pozzolanicity or its behaviour as a filler reported in the literature [15], nor of the jagged shape of recycled aggregates [74]. Be it said with respect to the latter that the respective interlocking behaviour is expected to be lower in material prone to shear failure [75], a characteristic likewise typically observed in concretes with recycled aggregates. According to Yang et al. [69], flexural strength declines may be attributed to the water absorption and oven-dry density values of the recycled aggregates used, which have particularly adverse implications for this parameter.

To date, very few studies have been published on the effect of including both recycled materials to replace gravel and conventional cement on concrete flexural strength. Cantero et al. [14], however, in research conducted under the same conditions as this study (i.e., 50% mixed RA and 25% brick powder), observed a 16.8% decline in 28 d and a 17% decline in 90 d recycled concrete strength relative to the reference. Those findings were similar to the results recorded by Letelier et al. [13], who limited the masonry addition to 15% and the recycled aggregate from precast concrete unit waste (normally deemed to be of higher quality) to 30%.

**Figure 4:**
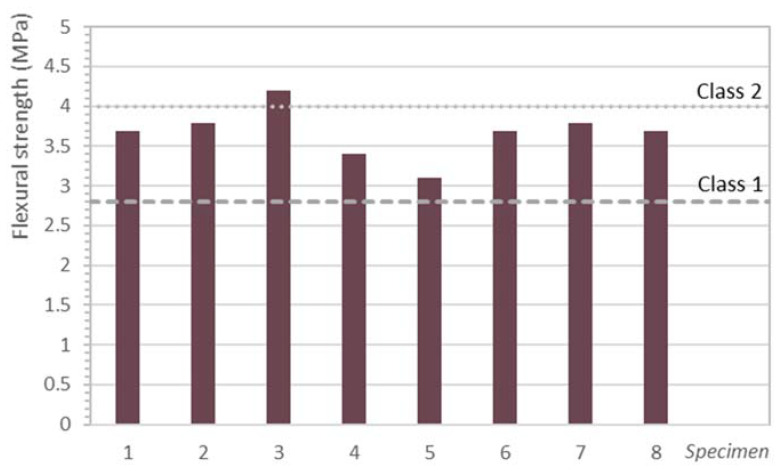
Flexural strength in eight RACC kerbs and minima specified in standard EN 1340.

Figure 4 graphs the results for the eight kerbs tested to failure as per standard EN 1340 [46,47], along with the minimum values to qualify as class 1 (dashed line) and class 2 (dotted line) materials. Further to those data, the RACC precast units were classified under class 1, for none of the values for the individual units tested was lower than 2.8 MPa and its characteristic flexural strength was over 3.5 MPa (Table 9). The kerbs would therefore qualify for product marking ‘S’, indicating aptness for use in pedestrian or light vehicle traffic. The flexural strength values found by Cantero et al. [14], at 5.64 ± 0.21 MPa, would indicate that RACC kerbs might even meet the class 2 requirement, with the concomitant broadening of their range of application. The Cantero et al. [14] findings for the replacement ratios in dually substituted concrete are consequently regarded as very promising for the valorisation of that fraction of CDW.

#### 3.2.3. Paving Block Splitting Tensile Strength

Further to a very thorough review of the literature conducted by Silva et al. [76], the replacement of natural gravel with recycled coarse aggregate has been extensively shown to induce a decline in splitting tensile strength. Some authors have nonetheless reported improvements in that parameter [63,69,77,78,79,80,81,82], attributed to the stronger bond or ITZ between the rough surface of recycled aggregate and cement paste.

Ge et al. [55], exploring the effect of masonry additions on splitting tensile strength, found performance to be similar to the reference at replacements of 10% while observing declines in strength at ratios of 20% and 30%. Strength values of 2.5 MPa to 4 MPa strength values were nonetheless recorded thanks to the shape, surface texture and specific surface of the masonry powder. Olofinnade et al. [53] also observed steeper declines in splitting tensile strength with rising replacement ratios, ranging from 10% to 40%.

Further to the data in Table 9, the paving blocks tested here for splitting tensile strength yielded values of 3.9 MPa (CC) and 3.4 MPa (RACC). The use of 50% mixed recycled aggregate to replace conventional gravel, together with the addition of 25% masonry powder to the recycled cement, was therefore observed to have an adverse effect, with a decline on the order of 12.8% in recycled concrete performance. Such a narrow difference in mechanical strength may be attributed to a number of factors:

- the pozzolanicity of the recycled materials used [15], along with their filler effect;
- the greater specific surface of the brick powder applied (5735 cm^2^/g) than of CEM III/A (3593 cm^2^/g), which affects the strength of the bonds that can be developed [55,83];
- the absence of any technique to offset water absorption, lowering the effective w/c ratio and favouring higher RACC mechanical performance;
- the strong bond/ITZ between the recycled aggregate and the cement paste, as per the microstructural analysis discussed below.

Cantero et al. [14], working under the same conditions as applied here, (i.e., 50% recycled aggregate and 25% brick powder), observed a 23.4% decline in 28 d and a 28.7% drop in 90 d splitting tensile strength. The authors also reported that whilst with the use of up to 25% RA the 28 d decline was similar (24.8%), the value for the 90 d materials prepared with that ratio was a smaller 15.4%. In subsequent research, Cantero et al. [84,85] nonetheless observed declines of 48.2% relative to the conventional concrete. Their experimental design enabled these authors to distinguish between the effects of each replacement. Whereas a 25% cement addition lowered strength by 19.9%, the application of 50% RA led to 17.5% lower values. The authors consequently recommended that for 50% RA replacement, cement additions should be limited to 10% to elude adverse effects on splitting tensile strength, where they found performance to decline by 20.8%. Consequently, although in the present study aggregate replacement was observed to a lower splitting tensile strength, the magnitude involved may be deemed to be in line with the Cantero et al. [84,85] recommendation.

Based on the admittedly short number of studies, such as the one described in this article conducted to date, the quality of the RA used may be expected to have an uneven effect on recycled concrete splitting tensile strength. That observation was reported in earlier studies on the separate use of recycled aggregate from CDW. Sanchez and Alaejos [86] found the values of the difference relative to the reference concrete to be reported by different researchers to range by ±20%, attributing that wide scatter to the variability in the recycled aggregate used. A factor of particular significance in connection with the recycled aggregates used in this study is that as they were applied directly as supplied by a CDW management plant with no laboratory pre-conditioning, the results are indicative of strength values that may be expected in actual practice.

Although, as noted earlier, the decline in recycled concrete performance may be deemed to be of scant quantitative significance, the adverse effect still gives cause for concern, because of the precast units made here, only those prepared with CC met the EN 1338 [42,43] minimum characteristic strength requirement (3.6 MPa). From that perspective, the values published by Cantero et al. [84,85] for concretes with the same replacement ratio as those studied here would not be standard-compliant, although neither would the conventional concrete used. Nonetheless, in an earlier study, the same authors [14] found both the conventional and the recycled concretes (bearing 50% RA and 25% brick powder) to exhibit values higher than those laid down in the standard. Although the effect of the quality of recycled aggregate on the end mechanical strength of the recycled concretes must not be underestimated, the explanation for that divergence would appear to lie more in the 100 kg/m^3^ difference in cement content between the two studies and the lower w/c ratio applied in the earlier research [14]. The inference is that concrete batching may be varied to meet the 3.6 MPa requirement with no need to forgo the dual reuse of mixed recycled aggregate from CDW in precast paving block manufacture.

**Figure 5:**
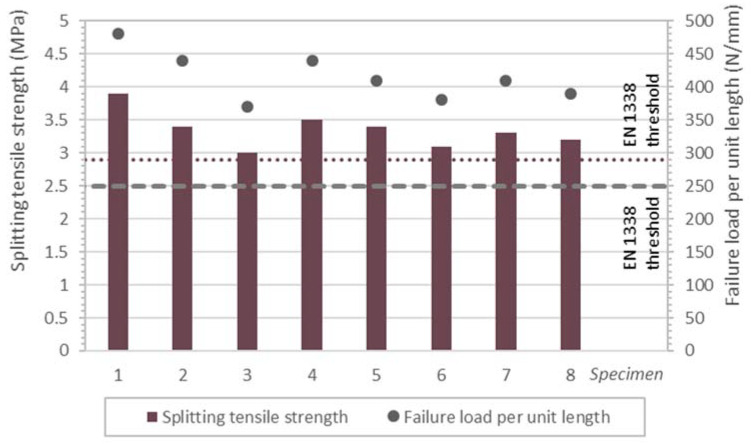
Splitting tensile strength in eight RACC paving blocks and minima specified in standard EN 1338.

European standard EN 1338 [42,43] also stipulates that no individual characteristic strength result may be below 2.9 MPa, nor may the failure load per unit length be less than 250 N/mm. The former threshold is shown as the dotted line and the latter as the dashed line in Figure 5, which graphs the individual findings for the eight paving blocks tested for splitting tensile strength as per EN 1338 [42,43]. While all the specimens met both requisites, the margins varied: whereas the failure load per unit length was comfortably met by all the samples, with a mean of 415 N/mm for the ones prepared with the RACC, the margin over the minimum characteristic strength was narrower, particularly in specimens 3 (3 MPa) and 6 (3.1 MPa).

#### 3.2.4. Abrasion Resistance

**Figure 6:**
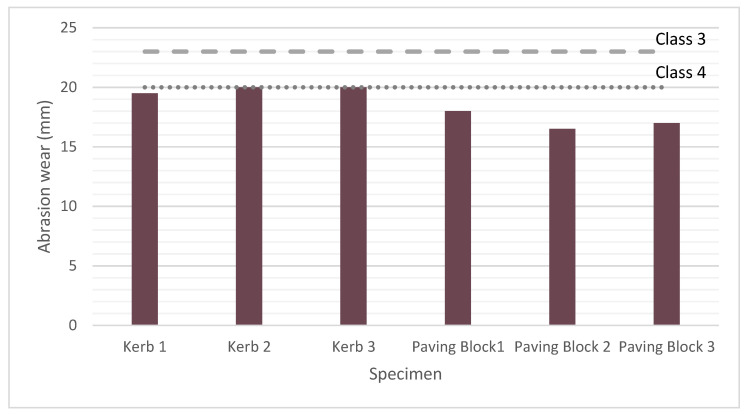
Abrasion resistance test findings for six RACC specimens (three kerbs and three paving blocks) and maxima specified in the respective standards, EN 1340 [46,47] and 1338 [42,43].

The abrasion resistance test conducted on three kerbs and three paving blocks showed that including recycled cement and aggregates in the mixes delivered high quality products in both cases. Although fairly tightly, the kerb values (19.8 ± 0.3%) met the class 4 (product marking ‘I’) requirement, the most demanding was defined in standard EN 1340 [46,47]. Standard EN 1338 [42,43] on paving blocks specifies the same criteria for abrasion resistance classes 3 (≤23 mm, product marking ‘H’) and 4 (≤20 mm, product marking ‘I’), the mean value of which is 17.2 ± 0.8%. As the bars in Figure 6 show, the three samples tested were comfortably below the most restrictive maximum value. Both types of units prepared with recycled concrete (bearing both recycled aggregate and recycled cement) therefore conformed to the very demanding requirements laid down for class 4, product marking ‘I’. The values recorded for these products also improved on the performance observed for the control concrete samples, CC, which according to manufacturer specifications were classified as class 3, product marking ‘H’, with mean resistance to wear of 23 mm. That improvement might be due to the strong recycled aggregate-paste ITZ developed in this type of recycled concretes. Furthermore, recycled aggregate, while not as mechanically strong as natural aggregate in terms of wear on individual particles (Los Angeles test), responds differently to abrasion when in the concrete matrix. Natural aggregate resists abrasion well until wear on the aggregate-paste ITZ reaches a certain point, after which the particles separate off the paste completely. In contrast, recycled aggregate, particularly when sourced from masonry waste, but also when made from recycled concrete, tends to wear gradually without detaching entirely, behaviour that translates into more effective abrasion resistance.

Authors such as Mas et al. [30] also tested recycled concretes where 20% or 40% of the natural coarse material was replaced by recycled mixed aggregate, reporting that abrasion resistance in the concrete with 20% replacement was equivalent to the value found for the control, although it dipped slightly when the ratio was 40%. The authors observed that during the test the abrasion wheel spontaneously pivoted to elude the masonry particles. They attributed that behaviour to the extremely hard composition of some of the masonry products which, while more brittle than natural aggregate, feature stronger surfaces. Brito et al. [87] mentioned the same instrumental behaviour. In another study, Rodriguez et al. [7] prepared terrazzo for indoor flooring, hollow tiles, kerbs and paving blocks, replacing 25%, 50%, 57% or 100% of the natural coarse with mixed recycled aggregate. They concluded that in the terrazzo tiles, all the recycled materials exhibited abrasion resistance similar to the precast units manufactured with the reference concrete. In the paving blocks and kerb units, however, 100% replacement lowered performance, although the recycled products featured the same resistance as the reference samples at ratios of up to 75%. Jankovic et al. [2], replacing both the fines and the coarse aggregate with brick masonry waste at different rates in paving blocks and flags, observed precast unit abrasion resistance to decline with rising recycled aggregate content, although all the paving blocks tested met the minimum requirements laid down in European legislation.

#### 3.2.5. Electrical Resistivity

**Figure 7:**
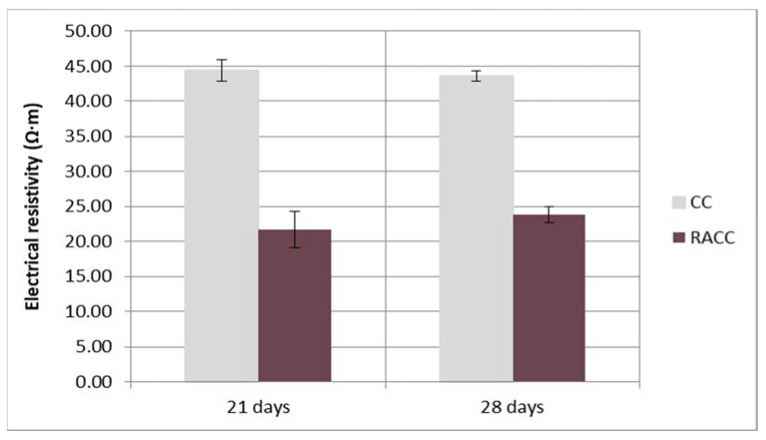
Electrical resistivity in 21 d and 28 d CC and RACC samples.

The 21 d and 28 d electrical resistivity observed for the control and recycled specimens (Figure 7) show that the joint use of the two types of recycled materials (coarse aggregate and cement powder replacements) had an adverse effect on this property. The declining values at both ages theoretically inferred lower concrete durability. That behaviour can be attributed to the predominance of the effect of recycled aggregate, one of the factors with the greatest impact on resistivity according to Hou et al. [88], who suggested that electrical resistivity of concrete is sensitive to variations in coarse aggregate content, size and type. A number of other authors have reported similar findings when raising the coarse aggregate replacement ratio in concrete [89,90,91,92]. In contrast, replacing portland cement with the masonry fraction of CDW has been shown to increase electrical resistivity and the protection it affords against corrosion [93,94,95]. That effect might be explored in future research, raising the recycled brick powder content and lowering the proportion of recycled aggregate, for instance, or combining the masonry addition to the cement with other types of recycled aggregates featuring other characteristics. Authors such as Medina et al. [96], using recycled sanitary ware waste, and Portella et al. [97], secondarily recycling insulator porcelain waste to replace natural aggregate, observed higher electrical resistivity in such recycled concrete rather than in conventional concrete.

Further to the pattern visible in the values observed for the samples (Figure 7), electrical resistivity tended to rise with curing time in the RACC samples, whilst the opposite effect was observed in the CC sample, where it tended to decline over time. As a result, the substantial (51%) difference between the two 21 d specimens narrowed by six percentage points (to 45%) just one week later.

#### 3.2.6. Water Absorption

**Figure 8:**
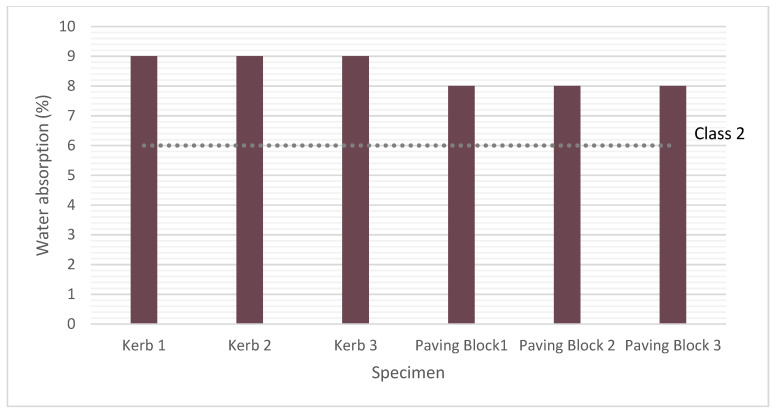
Water absorption test findings for six RACC specimens (three kerbs and three paving blocks) and maxima specified in the respective standards, EN 1340 [46,47] and 1338 [42,43] for class 2/product marking ‘B’ classification.

Water absorption is one of the most difficult parameters to control when working with recycled aggregate due to its higher sorptivity than found in the natural material. As the bars in Figure 8 show, all the paving blocks and kerbs prepared with recycled cement and aggregate exhibited values higher than the 4% mean recorded for the CC samples, being 9% the mean valued for kerbs and 8% for paving blocks. According to those findings, the recycled precast concrete units made with RACC did not qualify for class 2 (product marking ‘B’) status, which requires water absorption no greater than 6%, although they proved eligible for a class 1 (product marking ‘A’) classification, for which no performance criterion is stipulated.

The adverse effect of recycled aggregate on water absorption in precast concrete units has been observed by earlier authors. In a study jointly replacing different proportions of natural fine and coarse aggregate with brick masonry waste in paving blocks and flags, Jankovic et al. [2] detected the same problem: all the recycled concrete samples tested exceeded the class 2 value specified in the standard. Nonetheless, the test results after 28 freeze–thaw cycles indicated paving block and flag compliance with class 3 (weight loss of ≤1.0 kg/m^2^) weathering resistance requirements. Sadek and Nouhy [4], who also used crushed masonry to manufacture paving units with different proportions of masonry coarse or fine aggregates or both, reported water absorption rates higher than those allowed in the legislation, although the absorption recorded for the mixes with recycled fines was similar to the values observed for the control samples. El-Kattan et al. [98], in turn, using masonry waste to replace 1 wt%, 3 wt% or 5 wt% white cement, found water absorption to rise with the replacement ratio in all cases. Pentado et al. [6], however, applying ceramic tile polishing waste as a replacement for cement and fines in paving block manufacture, reported a decline in water absorption due to the filler effect. They contended that paving blocks able to support heavy vehicle traffic can be produced, provided the fines replacement ratio is limited to 30% and the cement replacement to 20%.

#### 3.2.7. Microstructure

**Figure 9:**
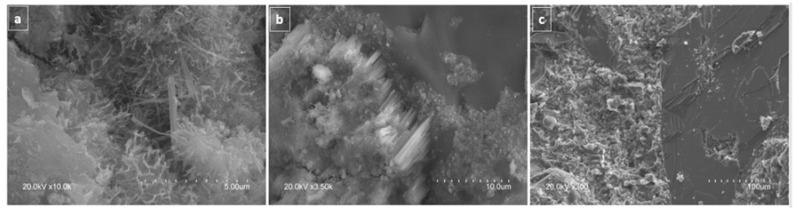
SEM micrographs of RACC: (**a**) calcium silicate hydrate gel honeycombing and ettringite needles; (**b**) column-like clusters of portlandite; (**c**) portlandite plates.

The structures characteristic of cement hydration products such as portlandite and CSH gels (honeycombed calcium silicate hydrate gel, ettringite needles, portlandite in column-like clusters and plates) are readily recognisable in the SEM micrographs of the RACC concrete reproduced in Figure 9. The inference is that portland cement replacement with masonry powder did not obstruct normal cementitious product hydration in the concrete mixes prepared.

Similar results were published by other researchers such as Bignozzi and Saccani [99], who explored masonry waste both as an aggregate and a supplementary cementitious material. Their findings showed no significant differences in the morphology of the matrices imaged. A study by El-Kattan et al. [98] of the 90 d morphology of white cement replaced at ratios of 1 wt%, 3 wt% or 5 wt% showed that the samples containing the masonry waste were more compact than the controls. The authors attributed that development to the formation of a silicate network and an excess of hydration product.

#### 3.2.8. Porosity

**Figure 10:**
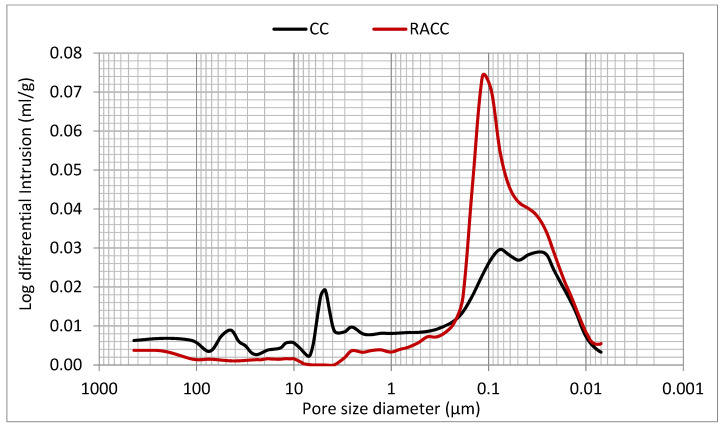
Pore size distribution—Log differential intrusion in 28 d CC and RACC samples.

**Figure 11:**
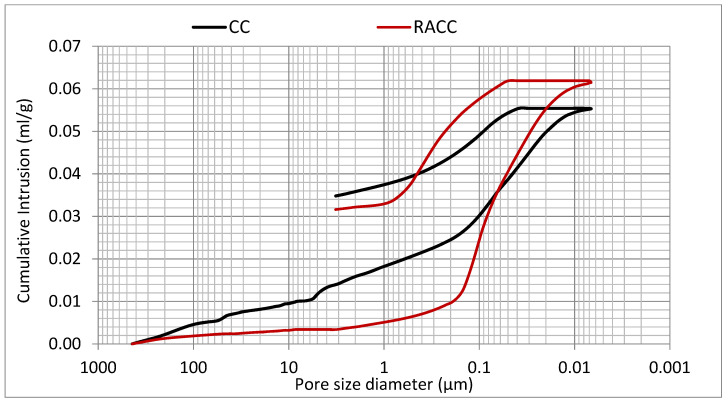
Pore size distribution—Cumulative intrusion in 28 d CC and RACC samples.

According to the log differential intrusion-measured pore diameter (mL/g)/pore size (µm) and ore size distribution (mL/g)/cumulative intrusion (µm) curves for CC and RACC in Figure 10 and Figure 11, the inclusion of masonry waste aggregate and especially masonry powder as a cement replacement lowered the proportion of pores with diameters greater than 0.2 µm, filling specially mesopores and macropores (pore size higher than 0.1 µm) [100,101] and, consequently, improving their resistance to penetration on attack by external agents. That observation, likewise reported by other authors [1,93,102], is attributable to the effect of masonry powder microfilling. When El-Dieb and Kannan replaced 10%, 20%, 30% or 40% of conventional cement with masonry powder, they recorded declines in all permeable pore volumes. Kannan et al. [94] also stressed the beneficial effect of the use of masonry waste powder on concrete manufacture. They noted, however, that while the effect was substantial up to 20% replacement, from 30% and higher the difference from the conventional material in permeable pore volume, although still favourable, tended to narrow. Asensio de Lucas et al. [32] analysed the use of masonry construction and demolition waste as a pozzolanic addition in blended cements, observing that the concretes prepared with the resulting binder had a more refined pore structure, potentially improving concrete durability.

At pore sizes of 0.2 µm and smaller, the voids volume was considerably greater in the RACC than in the CC (Figure 10), due primarily to the presence of recycled coarse aggregate. That finding was also reported by other authors [81,103]. Nonetheless, other studies [100,101] have shown that pore sizes of under 0.1 µm have the greatest impact on concrete unit durability. Such increases in the volume of pores of around that size in the RACC sample do not therefore infer poor kerb and paving block performance.

## 4. Conclusions

The experimental investigation carried out in this paper showed that the use 50 wt% mixed recycled aggregate (masonry content of 33%) and an eco-efficient cementitious material as 25 wt% conventional binder replacement is a technically viable option, as well as and European standard-compliant, for the manufacture of recycled precast elements (kerbs and paving blocks) used in footways.

The conclusions drawn include the following:

1. Although no significant textural differences were detected between the CC and RACC specimens, highly visible variations in colour were perceivable, as the inclusion of masonry CDW lent a reddish tone to the RACC units. In any case, no cracking or surface flaking was observed in any of the specimens prepared.
2. The strong recycled aggregate-cement paste ITZ, the non-compensation of water absorption by recycled materials and the pozzolanicity and greater specific surface of those materials relative to ordinary cement were identified as positive factors contributing to the mechanical strength of the RACC-mixture. Although early age mechanical strength was lower in the RACC than in the CC, the 28 d compressive strength was 37.5 MPa in RACC, values comfortably higher than the batching target, and 6% higher in RACC than in the CC. Nonetheless, RACC precast units exhibited a 26% lower flexural strength and a 12.8% lower splitting tensile strength compared to the conventional kerbs and paving blocks (CC-concrete), although the variation was much narrower than that reported in the literature.
3. The product marking classification of the recycled kerbs and paving blocks according to the European standards was also used in the comparison to the conventional precast products. On one hand, as a result of the strong recycled aggregate-cement paste bond and the higher abrasion resistance of the recycled coarse aggregate, the recycled precast elements were compliant with the strictest European criterion (class 4, ‘I’) whereas those of CC-concrete qualified for class 3 (product marking ‘H’). On the other hand, recycled kerbs and paving blocks were classified under class 1/product marking ‘A’ as the 6% water absorption limit was exceeded; thus, it was a less favourable category than that obtained by the conventional precast elements.
4. Regarding the electrical resistivity, an overall lower performance (51% at 21 days and 45% at 28 days) was noticed for RACC concrete. Nonetheless, as the literature suggests, the use of different replacement ratios and/or types of recycled materials might mitigate the adverse effect of CDW on this property.
5. In terms of microstructure, the use of recycled aggregate and masonry additions did not alter the formation of portlandite, CSH gels or ettringite and no risk of hydration reactions was identified. Moreover, the higher volume of pores with diameters of under 0.2 µm noticed in the RACC compared to CC due to the presence of recycled coarse aggregate does not compromise recycled concrete durability but reduces the number of pores larger than 0.2 µm, potentially improving concrete unit durability by refining pore structure.

## Figures and Tables

**Table 1:** Chemical composition of natural fine and coarse aggregate.

Component	SiO_2_	Al_2_O_3_	Fe_2_O	CaO	MgO	K_2_O	TiO_2_	MnO	CuO	ZrO_2_	LoI *
Fine	95.31	2.24	1.06	0.16	-	0.38	0.17	0.05	0.03	0.03	0.57
Coarse aggregate	98.09	1.17	0.27	0.05	-	0.14	-	-	-	-	0.28

* LoI: loss on ignition.

**Table 2:** Physical and mechanical properties of mixed recycled aggregate.

Properties	Test Result	Limit Value	Standard Applied
Maximum particle size (mm)	20	-	Spanish Structural Code [16]; EN 933-1 [18]
Minimum particle size (mm)	4	4	Spanish Structural Code [16]; EN 933-1 [18]
D/d ratio	5.0	≥1.4	Spanish Structural Code [16]; EN 933-1 [18]
Granulometric modulus	7.67	-	UNE 146406 [19]
Undersize particle content (%) Sieve d	5.03	<10	UNE 146121 [20]; EN 933-1 [18]
Oversize particle content (%) Sieve 2D	0	0	UNE 146121 [20]; EN 933-1 [18]
Oversize particle content (%) Sieve D	2.21	<10	UNE 146121 [20]; EN 933-1 [18]
Fines content (%)	0.04	≤1 (UNE 146121)	UNE 146121 [20]; EN 933-1 [18]
Apparent density (Mg/m^3^)	2.53	-	EN 1097-6 [21]
After oven-drying density (Mg/m^3^)	2.08	-	EN 1097-6 [21]
Saturate surface density (Mg/m^3^)	2.26	-	EN 1097-6 [21]
Water absorption (%)	8.53	≤7	Spanish Structural Code [16]; EN 1097-6 [21]
Flakiness index (%)	14.75	≤35	Spanish Structural Code [16]; EN 933-3 [22]
Los Angeles coefficient (%)	40.99	≤40–50 ^(1)^	Spanish Structural Code [16]; EN 1097-2 [23]

^(1)^ Bulk and reinforced concrete with characteristic strength of not over 30 N/mm^2^ (the concretes prepared here were compliant with that specification) can be made with coarse aggregate exhibiting a Los Angeles coefficient (abrasion resistance) of 40 to 50, providing its use is not detrimental to concrete properties based on prior experience and specific studies endorsing its aptness.

**Table 3:** Non-floating components in recycled aggregate.

Component	wt%
Concrete and mortar (natural aggregates with cement mortar attached)	44.11
Masonry (brick, roof tiles…)	33.56
Unbound aggregate (natural aggregate with no cement mortar attached)	17.51
Asphalt	0.44
Glass	0.75
Gypsum, wood, metals, plastic and other impurities	3.64

**Table 4:** Chemical composition of cement.

Component/Property	Value (wt%)	Limit
Clinker (SiO_2_, Fe_2_O_3_, Al_2_O_3_, CaO, MgO and SO_3_)	54	35–64
Blast furnace slag	41	36–65
Minor components	5	≤5
Loss on ignition	1.5	≤5

**Table 5:** Chemical composition of the masonry brick powder used in eco-efficient cement manufacture [31,32].

Oxide (wt%)	CaO	SiO_2_	Al_2_O_3_	SO_3_	Fe_2_O_3_	MgO	K_2_O	TiO_2_	P_2_O_5_	Na_2_O	SrO	Mn_2_O_3_	Cl^−^	Cr_2_O_3_	LoI *
Masonry brick powder	3.70	59.89	18.51	1.16	3.06	3.92	4.74	0.65	0.17	1.36	0.02	0.09	0.02	0.01	2.66

**Table 6:** XRF-determined chemical composition of eco-efficient cement.

Oxide (wt%)	CaO	SiO_2_	Al_2_O_3_	SO_3_	Fe_2_O_3_	MgO	K_2_O	TiO_2_	P_2_O_5_	Na_2_O	SrO	Mn_2_O_3_	Cl^−^	Cr_2_O_3_	LoI *
Eco-efficient cement	48.70	30.00	7.26	2.43	3.24	2.86	1.58	0.35	0.15	0.51	0.06	0.08	0.04	0.01	2.66

**Table 7:** Mechanical, chemical and physical characterisation of eco-efficient cement.

Property/Component	Blended Eco-Efficient Cement	EN 197-1 [25] Requirement	
2 day compressive strength (MPa)	22.1	≥20	EN 196-1 [24]
7 day compressive strength (MPa)	45.8	-	
28 day compressive strength (MPa)	56.4	42.5–62.5	
2 day flexural strength (MPa)	6.74	-	
7 day flexural strength (MPa)	8.02	-	
28 day flexural strength (MPa)	9.24	-	
LoI * (%)	2.66	≤5	EN 196-2 [37]
SO_3_ (%)	2.43	≤4.0	
Cl^−^ (%)	0.04	≤0.1	
Initial setting time (min)	165	≥60	EN 196-3 [38]
Final setting time (min)	251	-	
Soundness (mm)	1	≤10	

* Note to Table 5, Table 6 and Table 7: LoI = loss on ignition.

**Table 8:** Concrete batching (quantities per cubic metre).

Concrete Batching (Quantity per Cubic Metre)	CC	RACC
Water (L)	155.21	155.21
Effective water (L)	155.21	128.13
CEM III/A 42.5 N/SR (kg)	312.50	0
Recycled (masonry) blended cement (kg)	0	312.50
0/4 mm sand (kg)	96.98	96.98
0/5 mm sand (kg)	441.81	441.81
4/10 mm gravel (kg)	484.92	242.46
6/16 mm gravel (kg)	161.64	80.82
4/16 mm mixed recycled aggregate (kg)	0	323.28
W/c ratio	0.50	0.50
Effective w/c ratio	0.5	0.41

Note: CC-control concrete; RACC—recycled aggregate and cement concrete.

**Table 9:** Mechanical strength of conventional and recycled concrete.

Property	Age	CC	RACC
Compressive strength (MPa)	7 days	24.3 ± 0.2	19.7 ± 1.0
	21 days	30.4 ± 0.3	26.9 ± 0.6
	28 days	35.0 ± 0.7	37.1 ± 1.4
Kerb flexural strength (MPa)	28 days	5.0 ± 0.12	3.7 ± 0.38
Paving block splitting tensile strength (MPa)	28 days	3.9 ± 0.54	3.4 ± 0.28
